# Relationship between Blood Myostatin Levels and Kidney Function:Shimane CoHRE Study

**DOI:** 10.1371/journal.pone.0141035

**Published:** 2015-10-26

**Authors:** Shozo Yano, Atsushi Nagai, Minoru Isomura, Masayuki Yamasaki, Tsunetaka Kijima, Miwako Takeda, Tsuyoshi Hamano, Toru Nabika

**Affiliations:** 1 Department of Laboratory Medicine, Shimane University Faculty of Medicine, Shimane, Japan; 2 Department of Functional Pathology, Shimane University Faculty of Medicine, Shimane, Japan; 3 Department of Environmental and Preventive Medicine, Shimane University Faculty of Medicine, Shimane, Japan; 4 Department of General Medicine, Shimane University Faculty of Medicine, Shimane, Japan; 5 The Center for Community-based Health Research and Education (CoHRE), Shimane University, Shimane, Japan; Ohio State University Medical Center, UNITED STATES

## Abstract

**Objectives:**

Myostatin (MSTN), a member of TGF-β superfamily, is produced in the skeletal muscle to inhibit myocyte differentiation. MSTN expression is increased in the skeletal muscle in patients with chronic kidney disease (CKD), which may play a role in the pathogenesis of sarcopenia or in the protein energy wasting (PEW). This observation implies that the plasma MSTN level may be correlated with kidney function. Thus, we conducted a cross-sectional study to evaluate the association between the plasma MSTN concentration and the estimated glomerular filtration rate (eGFR).

**Subjects and Methods:**

Subjects were 781 participants of a health examination performed in a rural area in Japan. Among them, 124 subjects were selected by stratified random sampling according to eGFR. Creatinine clearance (ClCr) by the Cockcroft-Gault equation was used as a measure of kidney function. Plasma concentration of MSTN was determined by ELISA.

**Results:**

The plasma MSTN level was not different between men (3.42±1.61 ng/mL) and women (3.27±1.43 ng/mL). In a simple regression analysis, the MSTN level was significantly correlated with eGFR (r = -0.25, p<0.01) and ClCr (r = -0.20, p<0.05) but not with age and BMI. In a multiple linear regression analysis, the MSTN level showed a negative correlation with eGFR (standardized β = -0.31, p<0.01) and ClCr (standardized β = -0.35, p<0.01) under the adjustment with age, sex, BMI and LDL-C. Weak correlation was observed between the MSTN level and BMI / the serum LDL-C level. When the subjects were stratified into 4 groups according to eGFR, MSTN was significantly greater in the groups with the lowest and the 2^nd^ lowest eGFR (3.55±1.79 and 3.76±1.75 ng/mL, respectively) than the level in the group with the highest eGFR (2.77±0.85 ng/mL).

**Conclusion:**

Plasma MSTN level was elevated in an early stage of CKD, which could be involved in the progression of sarcopenia.

## Introduction

Nutritional status is a strong determinant of mortality, especially in patients with the chronic kidney disease (CKD). In CKD patients, loss of quantity and quality of the skeletal muscle progressively occurs, which is called sarcopenia [1]. Further, under the maintenance dialysis therapy, the patients often show loss of fat tissue in addition to sarcopenia, which is now called the status of protein energy malnutrition or of protein energy wasting (PEW). Although PEW imposes significant influence on the patients’ prognosis, the pathogenesis of PEW remains to be elucidated [2].

Recent advance in this field indicated that myostatin (MSTN) expression was increased in the skeletal muscle in CKD patients at both mRNA and protein levels [3]. MSTN induced degradation of muscle fibers and inhibited the protein synthesis in the skeletal muscle [4]. Moreover, administration of the anti-MSTN peptibody suppressed muscle wasting in a mouse model of CKD [5]. These findings suggested that MSTN played a pivotal role in the pathogenesis of PEW in CKD patients and can be a good therapeutic target [6].

The amino acid sequence of the mature MSTN, which is a member of the TGF-β superfamily [called growth differentiation factor (GDF)-8 as well], is 100% conserved between human, mouse, and rat. The precursor form of MSTN (~ 50kDa) is cleaved by BMP-1 family proteases to make the active form of MSTN, which is a disulfide-linked dimer of the C-terminal fragments (~ 25kDa). Knockout of the MSTN gene in mice elicited increase of the muscle mass and decrease of the fat mass [7]. A loss of function mutation in the MSTN gene caused a marked increase in the muscle mass, which was found in the Bergian Blue bull [8]. A mutation in this gene was found in humans, which resulted in hypertrophy of the skeletal muscle [9]. MSTN secreted from the skeletal muscle binds to the activin receptor IIB-ALK4/5 heterodimer to stimulate Smad and MAPK signal pathways whereas it inhibits the PI3K-Akt signal pathway [10]. Currently, MSTN is considered to inversely regulate the skeletal muscle mass through suppressing muscle atrophy *via* regulating transcription of autophagy-related genes and the ubiquitin-proteasome system and through inhibiting the differentiation of myoblasts and inducing apoptosis in myocytes [3–6, 11].

In spite of much evidence suggesting that MSTN played a key role in PEW observed in patients with CKD, so far, there is no evidence showing whether the plasma MSTN level is correlated with the kidney function. In this study, we thus examined the association of the plasma MSTN level with the kidney function under a cross-sectional study design employing community-dwelling individuals.

## Materials and Methods

### Subjects

This study was part of the cohort study (Shimane CoHRE Study) conducted by the Center for the Community-based Health research and Education, Shimane University. This study has been undertaken in collaboration with counties located in rural areas of Shimane Prefecture, Japan [12] [13]. Inclusion criteria of participants of the present study were all individuals aged 35 or older who took health check examinations conducted in Okinoshima-island in 2014. Although we invited all the resident according to the criteria, the participants in this study were mostly over 50 years of age due to advanced aging in the area. Exclusion criteria were having severe disorders such as advanced cancer and heart failure. Based on these criteria, a total of 781 subjects were assigned to this study. Among them, 124 subjects (58 men and 66 women) were selected by stratified random sampling according to the estimated glomerular filtration rate (eGFR). Briefly, eGFR was calculated as described below, and then 14–17 male and female subjects whose eGFR was close either to 30, 60, 75, or 90 mL/min were consecutively assigned. History of diabetes mellitus (DM) was obtained through an interview. Fasting blood glucose measured on site was not included in the criteria for the diagnosis.

### Ethics

Written informed consent was obtained from each participant. The study protocol was approved by the local ethics committee of Shimane University.

### Data collection

Plasma sample was separated within 30 min of the blood being drawn and kept frozen at −80°C until the measurement of MSTN. Plasma concentration of MSTN in fasting blood sample was determined by ELISA as followed by the manufacture’s instruction of Quantikine® (GDF-8/Myostatin Immunoassay, R&D systems Inc.). Briefly, to remove the pro-peptide form of MSTN, 100 μL of plasma samples were incubated for 10 min at room temperature with 50 μL of 1N HCl and neutralized with 50 μL of 1.2N NaOH/0.5M HEPES. The samples were then diluted with 200 μL of the calibrator diluent before the assay. Therefore, the final dilution of the plasma samples for the assay was 1:4. In this assay system, a monoclonal antibody specific for the mature MSTN was pre-coated onto a microplate, and the concentration was quantified by the sandwich ELISA technique using a microplate reader at 450 nm. The mean minimum detection limit for MSTN was 2.25 pg/mL. Serum sample served for biochemical measurements of triglyceride (TG), high density lipoprotein cholesterol (HDL-C), low density lipoprotein cholesterol (LDL-C) and creatinine (Cr) by standard enzymatic methods. HbA1c was determined by HPLC. The coefficients of variation (CVs) of all measurements were less than 7%.

eGFR was calculated by the following formula revised by the working group of Japanese Chronic Kidney Disease Initiative [14]. The formula is as follows;

eGFR (mL/min/1.73m^2^) = 194 × (serum Cr, mg/dL)^−1.094^ × age^−0.287^ × (0.739, if female).

Creatinine clearance (ClCr) calculated by the Cockcroft-Gault equation was also used as a measure of kidney function.

### Statistics

Data were expressed as mean ± SD. Because of a skewed distribution, the plasma MSTN was analyzed after logarithmic (log) transformation. Pearson’s correlation coefficient and one-way analysis of variance (ANOVA) were employed in univariate analyses between MSTN and other variables. Then, the multivariate linear regression analysis and Tukey’s test were conducted. All statistical analyses were performed with the IBM SPSS Statistic software (SPSS Statistics 22.0). Statistical significance was defined as *p<*0.05.

## Results

### Demographic data of the studied population

The baseline characteristics of the 124 participants (58 men and 66 women) are shown in Table 1. Plasma MSTN concentration showed a skewed distribution from 1.24 ng/mL to 10.64 ng/mL in our participants (the average was 3.34 ng/mL, see S1 Fig). Thus, MSTN was logarithmically transformed before the analyses described below. The plasma MSTN level was not different between men and women (Table 1). In addition, there was no difference in the MSTN level between subjects with DM (n = 13) and without DM (n = 111) (S2 Fig).

**Table 1:** Characteristics of the participants.

	Total (124)	Men (58)	Women (66)
Age (year)	72.9±6.2	73.7±6.2	72.2±6.2
Height (m)	1.56±0.09	1.64±0.06	1.50±0.05 *
Weight (kg)	57.6±10.2	63.5±8.0	52.5±9.1 *
BMI (kg/m^2^)	23.5±3.3	23.6±2.7	23.4±3.8 *
Cr (mg/dL)	0.90±0.77	1.02±0.31	0.79±1.0
eGFR (mL/min/1.73m^2^)	66.1±21.7	60.7±18.2	70.8±23.5 *
ClCr (mL/min)	65.8±25.1	62.6±21.2	68.6±28.0
LDL-C (mg/dL)	122±27	115±27	128±26 *
HDL-C (mg/dL)	57±14	55±15	59±14
TG (mg/dL)	111±59	121±65	102±53
FPG (mg/dL)	101±16	103±16	99±15
HbA1c (%)	5.9±0.4	5.9±0.4	5.9±0.4
MSTN (ng/mL)	3.34±1.51	3.42±1.61	3.27±1.43

Data were expressed as mean±SD. n.s; not significant.

*; p<0.01.

### Linear regression analysis for plasma concentration of MSTN

In a simple regression analysis, MSTN was significantly correlated with eGFR (r = -0.25, p<0.01) and ClCr (r = -0.20, p<0.05) (Table 2). Further, the negative correlation of the MSTN level with eGFR and ClCr was confirmed in a multiple linear regression analysis under the adjustment of age, gender, BMI and LDL-C (Table 3). A weak correlation of the MSTN level was observed with BMI as well as with the serum LDL-C level.

**Table 2:** Correlation of the plasma MSTN level with various parameters.

	r	p
Age	0.07	0.43
BMI	0.05	0.58
eGFR	-0.25	0.0045
ClCr	-0.20	0.025
LDL-C	0.10	0.26
HDL-C	-0.057	0.53
TG	0.067	0.46
FPG	0.061	0.50
HbA1c	0.07	0.44

**Table 3:** Multiple linear regression analysis on the plasma MSTN level.

	β	SE	standardized β	p	β	SE	standardized β	p
Age	-0.001	0.003	-0.018	0.858	-0.002	0.003	-0.064	0.548
Gender*	0.006	0.032	0.018	0.854	0.017	0.031	0.048	0.594
BMI	0.005	0.005	0.093	0.304	0.011	0.005	0.216	0.032
LDL-C	0.001	0.001	0.194	0.040	0.001	0.001	0.192	0.044
eGFR	-0.002	0.001	-0.307	0.003	-	-	-	-
ClCr	-	-	-	-	-0.002	0.001	-0.346	0.004

*; women: 0 and men: 1

### Kidney function and plasma MSTN

**Fig 1:**
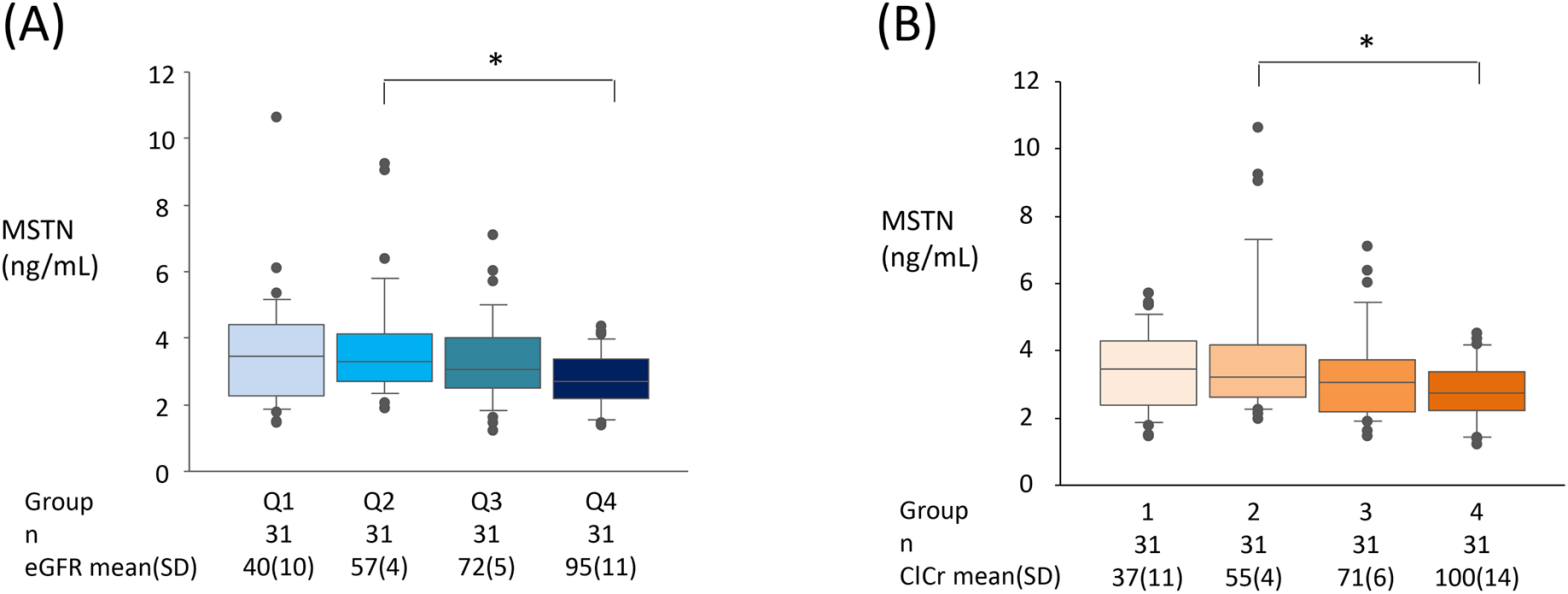
Relationship between the plasma MSTN and the kidney function. Box plots of plasma MSTN levels were shown in quartile groups categorized by (A) eGFR and (B) ClCr (see Methods). The upper, middle, and lower bars of the box represent 75%, 50% (median), and 25% of an each group. The upper and lower whiskers indicate the 90^th^ and 10^th^ percentile. The difference in the plasma MSTN level was examined among the quartile groups using one-way ANOVA. A significant difference was found in Q2 vs Q4 (*; p<0.05) by Tukey’s post-hoc test. eGFR and ClCr data were expressed as mean±SD.

Because we hypothesized that decrease in kidney function, even if it was modest, continuously affected the MSTN level, we simply categorized subjects into quartile groups for further analysis. When the subjects were stratified into quartile groups according to eGFR [between 4.1 and 50.7 mL/min/1.73m^2^ (Q1, n = 31), between 51.3 and 65.7 mL/min/1.73m^2^ (Q2, n = 31), between 65.8 and 78.6 mL/min/1.73m^2^ (Q3, n = 31), between 80.3 and 122 mL/min/1.73m^2^ (Q4, n = 31)], the MSTN levels in Q1 and Q2 (3.55±1.79 ng/mL; p = 0.20 and 3.76±1.75 ng/mL; p = 0.03, respectively) were greater than that in Q4 (2.77±0.85 ng/mL) (Fig 1A). This result was replicated even if quartile stratification was done using ClCr instead of eGFR; the MSTN level in Q2 (3.91±2.13 ng/mL) was significantly greater than that in Q4 (2.78±0.92 ng/mL) (Fig 1B).

These findings suggested that the plasma MSTN concentration was significantly elevated in an early stage of CKD. In addition, no matter which measure, eGFR or ClCr, was used as a parameter of kidney function, the MSTN level of the lowest quartile Q1 did not significantly differ from that of the 2^nd^ lowest quartile Q2, implying that elevation of the plasma MSTN in CKD subjects was not solely mediated by the accumulation due to impaired kidney function.

## Discussion

In this study, we found that the kidney function was independently correlated with the plasma MSTN concentration even in subjects with modest kidney dysfunction. As PEW or sarcopenia was not necessarily overt in those subjects, the present observation implied a potential physiological role of kidney in the regulation of MSTN [6].

Considering that the plasma MSTN level was a potential biomarker for sarcopenia and PEW [6], measurement of plasma MSTN may be clinically beneficial for CKD patients. In fact, Han et al. suggested that it was useful to measure MSTN in a late stage of CKD [15], and, in the present study, we extended this observation to patients with modest CKD. Although the mechanisms remain to be elucidated, reduction of the kidney function, even at a modest level, seems to induce the MSTN expression in the skeletal muscle, which may induce apoptosis of myocytes and inhibit the differentiation of myoblasts to myocytes [5, 6]. Therefore, even in patients with modest CKD, it may be useful to monitor the level of MSTN to obtain prognostic information concerning PEW. Moreover, neutralization of MSTN with and anti-MSTN antibody completely prevented the progression of muscle wasting in a CKD model animal, which implied that MSTN could be a good target for the treatment of PEW in CKD patients [5]. Finding of this study was an extension of the previous reports such as Verzola D. and coworkers [3]. However, we found that the plasma MSTN level might be a useful surrogate marker for sarcopenia, whereas Verzola et al. used the MSTN level in muscles, which was not easy to apply in clinical practice. Thus, this study retains new information that is clinically useful.

There are several reports concerning the blood MSTN level in humans [15–18]. Hittel et al. reported that ex-obese women showed a higher MSTN plasma level than that in lean women though they did not provide plasma concentrations of MSTN because they employed the Western blotting in the evaluation [16]. Using ELISA, Brandt et al. reported that the plasma MSTN level was 4–5 ng/mL in healthy subjects as well as in patients with DM [18], whereas Han et al. showed that two kits from different venders gave largely different values (32.5 μg/mL and 9.28 ng/mL when the sample from a healthy subject was measured) [15] [17]. In the present study, the averaged MSTN level was 3.34 ng/mL, which was relatively low when compared with those in previous reports. This discrepancy might be due to different procedures in the MSTN assay employed in the studies; for accurate measurement of the active form of MSTN, pretreatment of samples with acid or with heat is essential to remove the MSTN pro-peptide and other MSTN binding proteins [19, 20]. As most of previous studies did not describe whether they performed such pretreatment during the MSTN assay, it was possible that they had included inactive precursors of MSTN in their assay, which might give an apparent high level of MSTN. In spite that comparison was difficult among different studies because of the reason described above, the comparison among the individuals in this study was feasible as the measurement was performed using the same ELISA kit.

We found weak but significant associations of MSTN with LDL-C as well as with BMI, which implied a role of MSTN in the energy regulation and/or the lipid metabolism. This idea is supported by several animal studies; in Ldlr/Mstn double knockout mice, lack of Mstn expression reduced levels of VLDL, LDL-C, TG, and free fatty acid by 30–60% [21]. On the other hand, they may be concurrent events because an orphan nuclear receptor, Rev-erbbeta, simultaneously induced both the expression of Mstn and of genes involved in the lipid metabolism [22]. In humans, however, results of several studies were inconsistent with each other; the serum MSTN level was significantly reduced in individuals with the metabolic syndrome [15], while insulin resistance and BMI were associated with MSTN expression in the skeletal muscle in ex-obese subjects [16]. These observations including the present one suggested that extensive research is necessary to obtain a conclusive result in terms of a role of MSTN in the lipid metabolism. The positive association between BMI and plasma MSTN level is likely due to the positive association of skeletal muscle mass with BMI. However, it remains unclear whether the plasma MSTN level is generally correlated with the expression level of MSTN in the skeletal muscle.

In patients with DM, the MSTN expression was reported to be increased in the skeletal muscle but not in the plasma [18], while according to Han et al., the plasma MSTN level may be even lower in DM patients [15]. In contrast to these findings, we showed that the plasma MSTN level in DM patients was similar to that of non-DM individuals. Moreover, no significant association was observed between the plasma MSTN and HbA1c, which suggested that MSTN did not play a major role in the glucose homeostasis at least in our population.

There are several limitations in this study. First, this is a cross-sectional study, and thus, we cannot infer causal relationship between the MSTN level and the kidney function, LDL-C and BMI. Second, samples were selected by stratified random sampling. Although this sampling design might make the study more vulnerable to selection biases, it was expected that the level of MSTN was at least not biased by the selection because it was performed before measurement of MSTN. Third, since averaged age of our subjects was 72.9 years, the advanced aging might impact the results from the linear regression analysis of age and gender for plasma MSTN level. In spite of the limitations above, the present study provided an insight supporting a potential role of MSTN in the pathophysiology of sarcopenia, frailty and PEW in the elderly with modest CKD.

In conclusion, the present study indicated that the plasma MSTN level was elevated in early stages of CKD, which might contribute progression of sarcopenia in the elderly.

## Supporting Information

S1 Fig The histogram of the plasma MSTN concentration in the participants. Plasma MSTN concentration showed a skewed distribution from 1.24 ng/mL to 10.64 ng/mL in our participants (the average was 3.34 ng/mL).
(TIF)

S2 Fig Comparison of the MSTN levels in individuals with and without diabetes mellitus (DM). There was no difference in the MSTN level between subjects with DM (n = 13) and without DM (n = 111).
(TIF)
