# Supplementary figures and images for: Relationship between Blood Myostatin Levels and Kidney Function:Shimane CoHRE Study
Source: PLoS One. 2015 Oct 26;10(10):e0141035. doi: 10.1371/journal.pone.0141035 (PMC4621051; doi:10.1371/journal.pone.0141035)

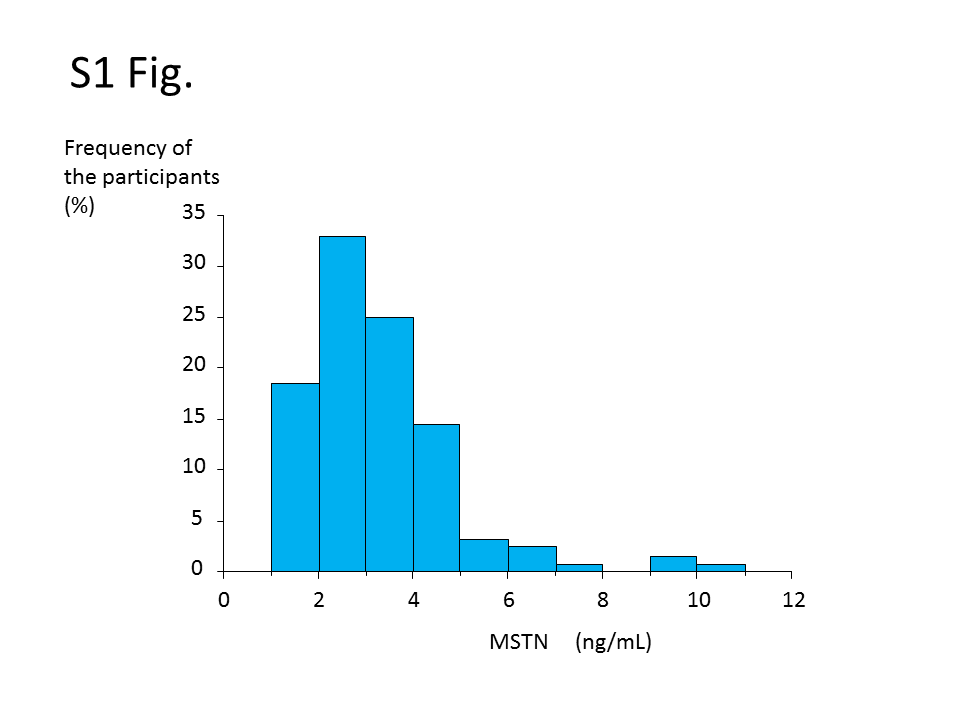

Supplement: S1 Fig — Plasma MSTN concentration showed a skewed distribution from 1.24 ng/mL to 10.64 ng/mL in our participants (the average was 3.34 ng/mL). (TIF) [file pone.0141035.s001.tif]

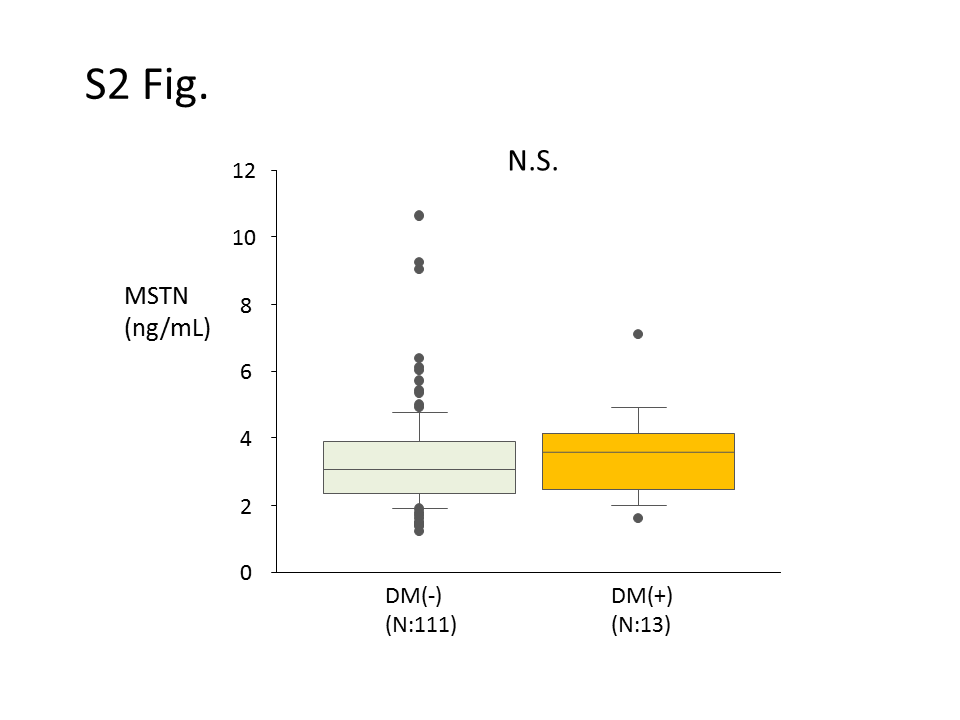

Supplement: S2 Fig — There was no difference in the MSTN level between subjects with DM (n = 13) and without DM (n = 111). (TIF) [file pone.0141035.s002.tif]
